# Restoration interventions mediate tropical tree recruitment dynamics over time

**DOI:** 10.1098/rstb.2021.0077

**Published:** 2023-01-02

**Authors:** Andy J. Kulikowski, Rakan A. Zahawi, Leland K. Werden, Kai Zhu, Karen D. Holl

**Affiliations:** ^1^ Environmental Studies Department, University of California, Santa Cruz, CA 95064, USA; ^2^ Lyon Arboretum and School of Life Sciences, University of Hawaii, Honolulu, HI 96822, USA

**Keywords:** forest, growth, seedling, sapling, survival, tropical

## Abstract

Forest restoration is increasingly heralded as a global strategy to conserve biodiversity and mitigate climate change, yet long-term studies that compare the effects of different restoration strategies on tree recruit demographics are lacking. We measured tree recruit survival and growth annually in three restoration treatments—natural regeneration, applied nucleation and tree plantations—replicated at 13 sites in southern Costa Rica—and evaluated the changes over a decade. Early-successional seedlings had 14% higher survival probability in the applied nucleation than natural regeneration treatments. Early-successional sapling growth rates were initially 227% faster in natural regeneration and 127% faster in applied nucleation than plantation plots but converged across restoration treatments over time. Later-successional seedling and sapling survival were similar across treatments but later-successional sapling growth rates were 39% faster in applied nucleation than in plantation treatments. Results indicate that applied nucleation was equally or more effective in enhancing survival and growth of naturally recruited trees than the more resource-intensive plantation treatment, highlighting its promise as a restoration strategy. Finally, tree recruit dynamics changed quickly over the 10-year period, underscoring the importance of multi-year studies to compare restoration interventions and guide ambitious forest restoration efforts planned for the coming decades.

This article is part of the theme issue ‘Understanding forest landscape restoration: reinforcing scientific foundations for the UN Decade on Ecosystem Restoration’.

## 1. Introduction

Ambitious efforts are underway to restore forests across the globe [1], yet restoration experiments at the temporal and spatial scale needed to guide these efforts are largely lacking [2]. Most tropical forest restoration studies and on-the-ground projects monitor only the first few years of recovery [3], despite the fact that achieving desired biodiversity and carbon sequestration benefits will take decades if not centuries [4]. Moreover, most past studies focus on measuring survival and growth of planted trees, whereas long-term success of forest restoration, particularly in highly diverse tropical forest, depends on the establishment, survival and growth of naturally recruiting trees. An extensive body of literature demonstrates the importance of quantifying demographic processes to understand community dynamics and successional trajectories in naturally regenerating (e.g. [5,6]), actively planted [7] and intact forests (e.g. [8,9]). However, rarely have survival and growth of recruiting tree seedlings and saplings been compared over time across different restoration approaches (but see [10]) ranging from passively (i.e. natural regeneration) to actively (most commonly planting trees) restored forests [11].

As politicians, investors, practitioners and the general public jump on the global bandwagon to plant a trillion trees worldwide, a repeated question has been whether investing resources in tree planting actually accelerates forest recovery over the long term [12,13], given that large areas of forests across the globe have regenerated naturally without human intervention [14]. In fact, recent meta-analyses suggest that overall recovery of biodiversity, carbon sequestration and nutrient cycling are similar or may even be faster in passively compared to actively (restoration plantings) restored forests [15,16]. Given the paucity of long-term studies that directly compare multiple forest restoration approaches at the same site [3,16], however, syntheses primarily rely on data from active and passive restoration from different locations and studies. Moreover, these meta-analyses are biased towards more successful naturally regenerated sites; i.e. studies of passively restored sites are typically conducted where forest naturally regenerated with minimal intervention, whereas actively restored sites span a broader range of conditions, including locations where recovery is slow or non-existent [17]. Finally, despite calls for replication of restoration sites across a landscape, most studies focus on a single or a few sites [2,3].

Long-term studies that directly compare active and passive restoration approaches at multiple sites are critical to guide investments and to gauge expectations in large-scale forest landscape restoration initiatives. Here, we compared annual survival of yearly updated cohorts and growth of naturally recruiting tree seedlings and saplings under three restoration treatments (natural regeneration, applied nucleation and tree plantations) replicated at 13 sites distributed across the landscape. We asked how recruit demography changed over a decade. Natural regeneration and plantation-style planting have been widely employed as forest restoration approaches, whereas applied nucleation (i.e. planting trees in clusters or islands) has been suggested as an intermediate intervention strategy that helps to overcome common barriers to natural regeneration while simultaneously promoting a more heterogeneous ecosystem [18]. Our prior research shows that applied nucleation and plantations are similarly effective in increasing the abundance and diversity of tree recruits, as compared to natural regeneration [19]. But quantifying how patterns of survival and growth of these recruiting individuals change over time is key to predicting future successional trajectories [10].

We used our decade-long demographic data to disentangle the effects of restoration treatment and recruit size on seedling and sapling survival, as well as sapling growth, of both early- and later-successional species. We also explored how seedling survival and sapling growth changed as succession progressed. We hypothesized that survival and growth of early-successional recruits would be highest in the applied nucleation treatment due to intermediate canopy and understory cover and, in turn, light availability [20]. The plantation treatments had high canopy cover through most of the study period, and low light availability is a key factor limiting seedling survival and growth in tropical forests, particularly for early-successional species [6,21]. By contrast, recruits in the natural regeneration plots must overcome strong competition from 1 to 3 m-tall pasture grasses [22]. We therefore expected lower early-successional survival in natural regeneration, but faster recruit growth rates than actively planted treatments due to the high-light environment once recruits overtopped competitive grasses [10,23], particularly early in the recovery process. By contrast, we anticipated that survival of later-successional species would be similar across treatments and change less over time, given that later-successional species are adapted to tolerate variable light environments [24]. Finally, we hypothesized that growth and survival rates across treatments would converge over time due to increasing canopy cover in natural regeneration and applied nucleation plots [18].

## 2. Methods

### (a) Study region

This study was conducted at 13 approximately 1 ha sites spread across an approximately 100 km^2^ area between the Las Cruces Biological Station (LCBS; 8° 47′ 7″ N; 82° 57′ 32″ W) and Agua Buena (8° 44′ 42″ N; 82° 56′ 53″ W) in southern Costa Rica (electronic supplementary material figure S1). The forests in this region are at the boundary between Tropical Premontane Wet and Rainforest zones [25], range in elevation from 1100 to 1430 masl and receive mean annual rainfall of 3500–4000 mm, with a dry season from December to March. Mean annual temperature is approximately 21°C. All sites are separated by at least 700 m, and most are steeply sloped (15–35°). Soils are volcanic in origin, mildly acidic, low in P and high in organic matter [26]. The landscape surrounding the plots is dominated by cattle pastures and low-intensity, sun-coffee plantations, with old-growth and secondary forest fragments of varying sizes covering 28% of the study region [27]. All sites were farmed for at least 18 years, and most were burned once or twice after clearing, but not thereafter. Most sites had been used for a mixture of cattle grazing and coffee farming and, at the start of the study, were either dominated by one or a combination of three forage grasses, *Axonopus scoparius* (Flüggé) Kuhlm, *Pennisetum purpureum* Schumach and *Urochloa brizantha* (Hochst. Ex. A. Rich.) R.D. Webster; or hosted a mixture of grasses, forbs, and the fern *Pteridium arachnoideum* (Kaulf.) Maxon.

### (b) Experimental design

Five sites were established in 2004, five in 2005 and three in 2006. At each site we established three 0.25 ha (50 × 50 m) plots, each separated by a buffer of at least 5 m. Each plot received one of three randomized treatments: natural regeneration, applied nucleation or plantation. Plantations were uniformly planted with tree seedlings, whereas the applied nucleation treatment was planted with six tree clusters (or islands) of three sizes: two each of 4 × 4, 8 × 8 and 12 × 12 m (electronic supplementary material, figure S2). Tree island sizes were randomly arranged within each row and were separated by at least 8 m. Planting density was kept constant (approx. 2.8 m between seedlings); 313 trees were planted in plantation, 86 in applied nucleation and none in natural regeneration plots. We planted seedlings (20–30 cm tall) of four tree species that have high survival and extensive canopy development [28]. These included two natives, *Terminalia amazonia* (J.F. Gmel.) Exell (Combretaceae) and *Vochysia guatemalensis* Donn. Sm. (Vochysiaceae) and two naturalized softwoods, *Erythrina poeppigiana* (Walp.) Skeels and *Inga edulis* Mart. (Fabaceae) that are used widely in intercropping systems in Central America. In all plots (including natural regeneration), all herbaceous and woody vegetation was cleared to ground level at approximately 3 month intervals for the first 2.5 years to allow planted tree seedlings to grow above existing vegetation. Vegetation clearing ceased between 2007 and 2009 depending on when the site was initially planted. By the end of the study, tree canopy cover (based on densiometer measurements) had increased substantially in all treatments to more than 60% in natural regeneration and over 90% in applied nucleation and plantations (electronic supplementary material, figure S3a).

### (c) Data collection

We surveyed tree seedlings (at least 20 cm tall and less than 1 cm diameter-at-breast height (DBH)) and saplings (at least 1 cm and less than 5 cm DBH) annually during late June–early July of each year in all restoration plots. We did not record recruits less than 20 cm in height because it was impossible to reliably detect them among the dense understory cover of grasses and ruderal forbs in many plots. We started sampling in 2007–2009 (3 years after a plot was established and 6 months after understory clearing ceased) and continued through 2019. Tree seedlings were measured in 1 × 2 m quadrats, and saplings were measured in 2 × 4 m quadrats. Quadrats were grouped into four belt transects, one in each quadrant of the plot in natural regeneration and plantations, and six associated with each of the tree islands in applied nucleation (*n* = 16 natural regeneration/plantation, *n* = 30 applied nucleation, electronic supplementary material, figure S2). The applied nucleation treatment was sampled more intensively to quantify potential differences in recruitment between planted and unplanted areas (electronic supplementary material, figure S2). Since survival and growth analyses were focused at the plant level and hence not dependent on abundances, we did not standardize for sampling area differences between applied nucleation and other treatments. Each recruit was permanently tagged and survival was recorded annually in subsequent years. Height was recorded for seedlings at least 20 cm tall and less than 1 cm DBH. DBH of the largest stem was recorded for saplings with stem width of at least 1 cm and less than 5 cm DBH. For seedlings that transitioned to saplings, survival observations were included in the seedling dataset until recruits exceeded 1 cm DBH. Thereafter, observations were included only in the sapling dataset. The sapling dataset did not include survival observations for saplings that transitioned to small trees (over 5 cm DBH).

### (d) Data analysis

We focused analyses on naturally established tree recruits that dispersed into plots from the landscape. Given our emphasis on forest succession, we omitted recruits from planted species, three of which had very low recruitment; the fourth (*E. poeppigiana*) frequently recruited but recruits suffered more than 95% mortality within the first year [29]. We also omitted individuals that resprouted following previous agricultural activities (primarily *Citrus* spp. and *E. poeppigiana*).

We modelled seedling survival and sapling growth separately for early- and later-successional species. Tree successional stage (early or later) was determined using local knowledge and literature references. We consider later-successional species as those that are found either in the understory of mature forests or in a mix of mature and secondary forests, but that do not establish during the earliest stages of succession. Natural regeneration plots were excluded from later-successional recruit models due to very low numbers of later-successional recruits (42 total). We pooled all recruits for sapling survival models as the separate model for later-successional saplings did not converge due to sample size constraints (early-successional saplings: *n* = 502, later-successional saplings: *n* = 221).

To examine the effects of restoration treatment and year since restoration on seedling (*n* = 1286) and sapling (*n* = 723) survival of annually updated cohorts, we used generalized linear mixed-effects models (GLMMs) with a binomial distribution (lme4 package, [30]). We define year since restoration as the time a seedling recruited after vegetation clearing ceased at a given site. We included size measurements of recruits from the preceding year (height for seedlings, DBH for saplings) as recruit size has been shown to strongly affect survival [31,32]. We initially included the following random intercepts in all models: restoration site as a blocking factor, tree species to account for species-specific variation and plant identity to account for repeated measures on each recruit. We used intraclass correlation coefficients (ICC) to assess the level of within-group similarity and determine the influence of random effects on model estimates ([33]; performance package, [34]). For all survival models, intraclass correlation was small for site and plant identity random effects (ICC < 0.10). These random effects were therefore omitted from models, making tree species (ICC > 0.10) the only random effect included. These omissions did not change trends compared to models including all random effects but aided in model fit to test hypotheses.

Whereas we collected recruit data from 2007 to 2019, there was an insufficient number of recruits in 2007 to include those data in models. Moreover, an examination of seedling age and mortality showed that more than 75% of seedlings that died did so by age three years. We therefore omitted recruits that established at the end of the study (between 2017 and 2019) from survival models as these seedlings may not have had sufficient time to die and would have biased our temporal comparisons. Both conspecific and overall density dependence based on total stem abundance per quadrat (early- and later-successional species pooled) were poor predictors of recruit survival in our preliminary models, so we did not include negative density dependence in any final models.

We modelled sapling (*n* = 1203) growth as absolute change in DBH between each annual sampling period (DBH*_t_* – DBH_*t–*1_) predicted by restoration treatment, year since restoration, their interaction and sapling size (DBH_*t–*1_) using GLMMs with Tweedie (compound Poisson-Gamma) distributed errors (package glmmTMB, [35]). Tweedie distributions are generalized power law distributions for modelling continuous data with many zeros and provided a better fit and convergence than other distributions. For both growth models, intraclass correlation was small for site- and tree species-level random effects (ICC < 0.05), which were omitted, leaving individual plant identity (ICC > 0.075) as the only random effect. Our sapling growth data span from 2010 to 2019 as the number of recruits was too small for analysis in the first 3 years since restoration. We did not analyse seedling growth (height) due to our sampling design; we transitioned from height to DBH growth measures at greater than 1 cm DBH so a seedling height analysis would be biased towards recruits with slow growth.

We assessed model performance and assumptions in the R package DHARMa [36], which uses a simulation-based approach to create standardized residuals for GLMMs. We then examined plots of standardized residuals versus predicted values and quantile–quantile plots of deviation from a uniform distribution (see [36] for details), which both indicated reasonable fit for all models. We report marginal and conditional pseudo-*R*^2^ (*R*^2^_M_ and *R*^2^_C_, respectively) following Nakagawa & Schielzeth [37] (performance package, [34]) as an analogue of goodness of fit appropriate for GLMMs. Collinearity was assessed using variance inflation factor (VIF) values among fixed effects. No collinearity was found (VIF < 10) in any model. We tested the effects of predictor variables and their interactions using likelihood ratio tests based on comparisons of *χ*^2^ distributions between full and reduced models. We assessed the effects of treatment levels as well as their interaction with time since restoration using bias adjusted, estimated marginal means (predicted survival probability and predicted ΔDBH; hereafter referred to as ‘survival probability’ and ‘growth’, respectively) and estimated slopes for interactions (package emmeans [38]). When appropriate, we used Tukey *post hoc* multiple comparisons (package emmeans [38]) to determine significant differences (*p* < 0.05) between restoration treatment levels. When needed, continuous variables were scaled using *z*-scores to improve model convergence. All analyses were conducted in R v.3.6.3 [39].

## 3. Results

### (a) Recruit survival

**Figure 1.**
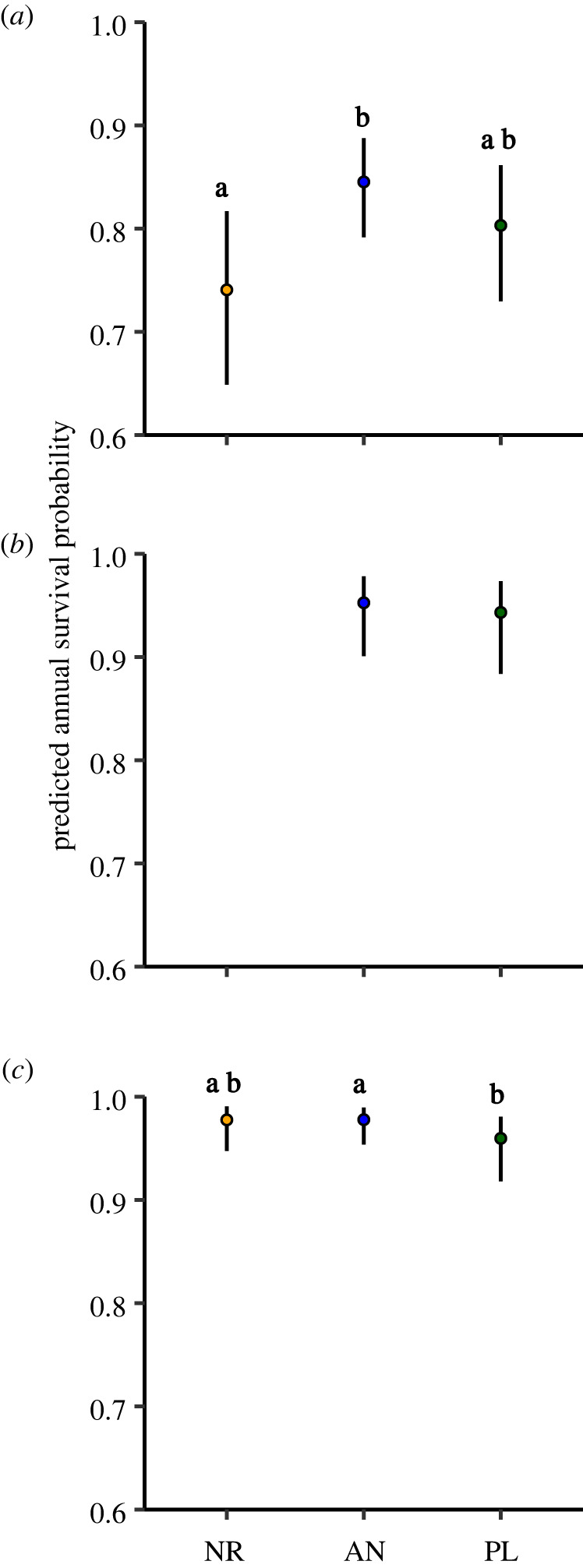
Predicted survival probabilities for (*a*) early-successional seedlings, (*b*) later-successional seedlings and (*c*) all saplings by restoration treatment. NR, natural regeneration (omitted for later-successional seedlings); AN, applied nucleation; and PL, plantation restoration treatments. Error bars are estimated 95% upper and lower asymptotic confidence levels around estimated marginal means. Means with the same letter do not differ significantly using Tukey's multiple comparison test among treatments. (Online version in colour.)

Early-successional seedlings had greater annual survival probability in applied nucleation than natural regeneration treatments (electronic supplementary material, table S1, *z* *=* 3.48, *p* = 0.001, *R*^2^_C_ = 0.22, *R*^2^_M_ = 0.12) but there was no significant difference between plantations and the other two treatments (figure 1*a*; electronic supplementary material, table S1, *p* > 0.19). As year since restoration increased, early-successional seedlings experienced marginally greater survival (electronic supplementary material, figure S3a, *p* = 0.07), but the treatment × year since restoration interaction was not significant (*p* = 0.86, electronic supplementary material, table S1). Later-successional seedling survival probability was similarly high (greater than 0.94) in applied nucleation and plantation treatments (figure 1*b*; electronic supplementary material, table S1, *p* = 0.44) and did not differ between treatments over time (electronic supplementary material, figure S3b and table S1, *R*^2^_C_ = 0.50, *R*^2^_M_ = 0.16). Taller seedlings of both early- (electronic supplementary material, figure S4a) and later-successional seedlings (electronic supplementary material, figure S4b) had higher survival (*p* < 0.001).

Sapling survival probability was very high for all three restoration treatments (figure 1*c*; electronic supplementary material, table S1, *R*^2^_C_ = 0.42, *R*^2^_M_ = 0.12), with slightly but significantly higher survival probability in applied nucleation compared to plantation treatments (*z* = 2.48, *p* = 0.04). Sapling survival probability decreased significantly with time since restoration (electronic supplementary material, figure S3c, *p* < 0.001) but stayed at high levels (greater than 0.96) throughout the study. Larger seedlings experienced higher survival (*p* < 0.001). By the end of the study, 87.2% of saplings had survived compared to 58.6% of seedlings.

### (b) Sapling growth

**Figure 2.**
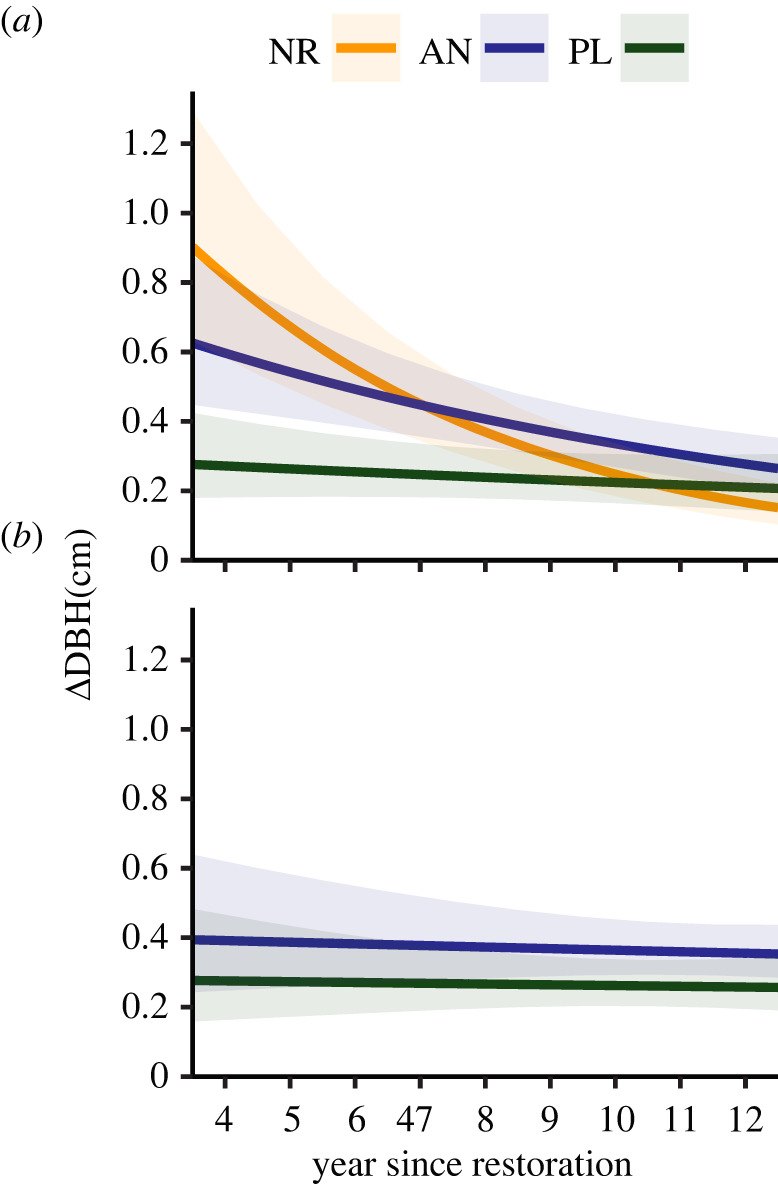
Predicted increase in growth (DBH) by year since restoration for (*a*) early- and (*b*) later-successional saplings in each restoration treatment. NR, natural regeneration; AN, applied nucleation; PL = plantation restoration treatments. Error bands are 95% confidence intervals around the estimated trend. (Online version in colour.)

**Figure 3.**
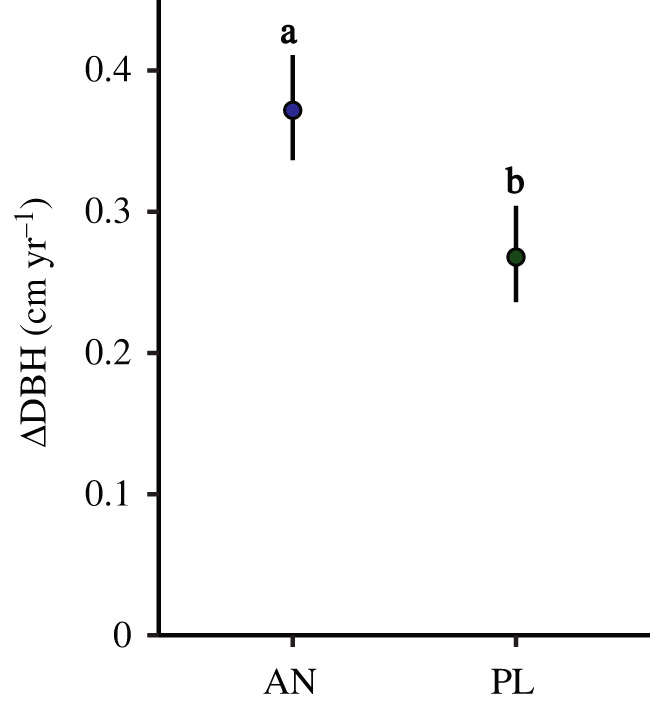
Predicted annual increase in later-successional sapling growth (DBH) in applied nucleation (AN) and plantation (PL) treatments. Error bars are 95% confidence intervals around estimated marginal means. Different letters represent a significant difference between treatments at the *α* = 0.05 level. (Online version in colour.)

Treatment and year since restoration had an interactive effect on early-successional sapling growth (figure 2*a*; electronic supplementary material figure S5a, *p* < 0.001), which reflects significant differences in estimated slopes of survival over year since restoration between all combinations of treatments (electronic supplementary material, table S1, *R*^2^_C_ = 0.79, *R*^2^_M_ = 0.14). In the first few years immediately following restoration, growth rates were much faster in natural regeneration and applied nucleation than plantation treatments. By the end of the sampling period, however, early-successional sapling growth had slowed substantially in applied nucleation and even more in natural recruitment plots, such that sapling growth rates had converged across treatments after nearly a decade (figure 2*a*; electronic supplementary material, figure S5a). By contrast, later-successional sapling growth remained relatively constant across the entire sampling period (figure 2*b*; electronic supplementary material, figure S5b, *p* = 0.46), but was significantly higher in applied nucleation than plantation treatments (figure 3; electronic supplementary material, figure S6, *p* < 0.001, *R*^2^_C_ = 0.63, *R*^2^_M_ = 0.04). Larger early-successional saplings grew faster (*p* < 0.001), whereas later-successional sapling growth showed no association with size (*p* = 0.57).

## 4. Discussion

Ours is the first long-term (10 year), multi-site study that compares survival and growth of naturally recruiting trees across a gradient of restoration interventions. Whereas many short-term studies (typically 1–5 years) comparing survival and growth of recruits show strong differences across restoration treatments (e.g. [40,41]), our results suggest that some differences among treatments may converge rapidly, even within the first decade. Nonetheless, we documented multiple differences in survival and growth of recruits across restoration treatments that could result in different rates of recovery, and possibly plant community composition, well into the future.

Lower survival of early-successional seedlings in natural regeneration plots (figure 1*a*) is likely explained by strong competition with non-native pasture grasses that many previous studies have shown to form a strong barrier to tree recruitment and survival (e.g. [22,42,43]). Indeed, grass cover was consistently higher in natural regeneration compared to other treatments after active management ceased, although it decreased over time (electronic supplementary material, figure S7b). Across all treatments, early-successional seedlings that established soon after restoration experienced marginally higher mortality than those arriving later in succession, suggesting that even modest levels of grass cover present before canopies close can affect early-successional seedling survival (electronic supplementary material, figure S3a).

Once seedlings survived to the sapling stage and hence overtopped non-native pasture grasses, survival was generally high, although slightly lower in the plantations than other treatments (figure 1*c*, [10]). Moreover, early-successional saplings grew faster in natural regeneration and applied nucleation treatments soon after restoration but growth rates declined and converged substantially a decade post-restoration (figure 2*a*). This result is consistent with many other studies showing that early-successional tree growth rates decline as light limitation increases [44,45]. Likewise, Caughlin *et al.* [10], who monitored growth 7 years post-restoration, reported that recruits reached reproductive maturity sooner in natural regeneration than plantation plots in Mexico. These patterns are likely due to the lower canopy cover and associated shading in the natural regeneration compared to plantation treatments (electronic supplementary material, figure S7a). Whereas other tropical forest succession studies have shown that seedling growth may be nutrient limited (e.g. [31,46,47]), surface soil nutrients in our sites were similar across treatments during the study ([26], K. Holl, R. Zahawi 2017, unpublished data). Additionally, litterfall inputs of nutrients most limiting to plant growth were highest in the plantation treatments early in this study [48], contrasting with lower growth rates in plantations compared to other treatments. Together these results suggest that sapling growth patterns in our sites are more driven by light than nutrient limitation.

Later-successional species showed similar seedling survival rates in applied nucleation and plantation treatments (figure 1*b*). This pattern is consistent with many prior studies demonstrating that later-successional, shade-tolerant recruits have higher survival overall and are less sensitive to light availability [24,49,50]. Moreover, seedling herbivory and subsequent mortality tend to be lower when comparing later-successional to early-successional seedlings [29], likely due to higher levels of secondary chemicals and mechanical defenses [51]. These results, as well as the fact that recruitment of later-successional seedlings in natural regeneration plots was so low that we could not compare survival, are consistent with past work of our group and others showing that dispersal-limitation is stronger than establishment-limitation for later-successional species in secondary tropical forests, particularly for the many species with large, animal-dispersed seeds [52–54].

We were surprised that the density of neither conspecific nor heterospecific species was a significant explanatory factor in our initial models, given that many studies have shown NDD to be an important factor affecting seedling survival in both naturally regenerating and intact natural forests [6,8,32]. We suspect that NDD was weak in our plots due to the fact that (i) we did not analyse recruit data from planted tree species and (ii) few recruited species fruited during the study period, so nearly all recruits came from outside the plots rather than falling in high density below fruiting trees. We anticipate that NDD will become more important over time as species within the plots become reproductively mature and note that further study of the role of NDD in seedling recruitment in a restoration context is needed.

A large body of literature shows that both recruit survival and growth rates typically increase with plant size and that the strongest filters to forest recovery act more strongly on smaller recruit size classes [9,10,55,56]. Our results are generally consistent with these patterns, with the exception of later-successional sapling growth rates which showed no correlation with initial sapling size (electronic supplementary material, table S1). Others have shown that late-successional saplings in the understory invest more in leaf replacement than other forms of growth [57]. Since all of our later-successional growth data come from plantation and applied nucleation treatments, high canopy cover in these treatments could explain the lack of a size effect on sapling growth [49,58].

Our results suggest that applied nucleation promotes an intermediate and more heterogeneous canopy cover [20,59], which in turn creates a light environment that facilitates survival and growth of both early- and later-successional tree recruits. Survival of both seedlings and saplings was similar in applied nucleation plots when compared to the more resource-intensive plantation forestry approach. Interestingly, later-successional sapling survival and growth were higher in applied nucleation than in the plantation treatment, which is supported by other studies showing that small-scale heterogeneity in light environments within closed canopy forests can drive growth dynamics of recruits in the understory [6,60]. In addition, previous studies of natural recruitment into older tree plantations show that medium-size classes of recruiting trees are underrepresented [61,62], whereas faster later-successional sapling growth in applied nucleation plots may ultimately lead to more heterogeneity in tree size classes. Together, these findings suggest that over the longer term, later-successional species may come to dominate our applied nucleation plots sooner than plantation plots, with implications for more rapid reassembly of mature tropical forest communities. However, we hasten to note that we only planted four species of trees, since our goal was to rapidly achieve canopy cover that enhanced the rate of succession by attracting dispersers and providing more favourable conditions for seedling establishment. The results of both applied nucleation and plantation restoration strategies would differ if later-successional species were included as part of the initial planting mix or actively planted after the initial canopy established. Indeed, the effect of species composition on the relative effects of applied nucleation and plantation planting strategies is an important area for future research [18,63].

The results presented here, combined with our prior work showing that applied nucleation facilitates later-successional seed dispersal to a similar degree to tree plantations [52], suggest that applied nucleation not only provides a promising and cost-effective approach towards overcoming initial barriers to tree establishment, but also facilitates the recovery of the forest composition beyond the first decade. However, the most appropriate restoration approach for a given site depends critically on the project goals and the ecological and social conditions [64]. Applied nucleation is likely to be an effective strategy to facilitate forest recovery in cases where there are sufficient sources of a diversity of tree seeds and seed-dispersing animals in the surrounding landscape; woody vegetation nuclei are lacking in the restoration site; spread of tree nuclei is not inhibited by herbivory, invasive species competition, or fire; and it is compatible with landowner preferences [18]. In regions with small landholders who depend heavily on income from their land, extensive planting of native species that provide fruit, firewood or timber resources will often be more appropriate. Likewise, extensive planting of a diversity of trees may be necessary in highly degraded sites that lack nearby seed sources. The next step is to implement the applied nucleation approach in on-the-ground restoration projects [63] at the scale of tens to hundreds of hectares and monitor outcomes [63], such as a new 500 ha project being undertaken by Conservation International in the Brazilian Amazon region (S. Sprenkle 2022, personal communication). Long-term, multi-site studies are key to determining under what conditions different restoration strategies are most effective to achieve the biodiversity and carbon sequestration goals of the ambitious forest restoration efforts planned for the coming decades.

## Data Availability

Data are available from the Dryad Digital Repository: https://doi.org/10.7291/D1H10M [65]. The data are also provided in the electronic supplementary material [66].
